# Insights into the mechanisms of microRNAs in hepatoblastoma: from diagnosis to treatment

**DOI:** 10.1093/pcmedi/pbaf034

**Published:** 2025-11-19

**Authors:** Meng Kong, Shisong Zhang, Xiang Ma

**Affiliations:** Department of Pediatric Surgery, Children’s Hospital Affiliated to Shandong University, Jinan 250022, China; Department of Pediatric Surgery, Jinan Children’s Hospital, Jinan 250022, China; Department of Pediatric Surgery, Children’s Hospital Affiliated to Shandong University, Jinan 250022, China; Department of Pediatric Surgery, Jinan Children’s Hospital, Jinan 250022, China; Department of Respiratory Disease, Children’s Hospital Affiliated to Shandong University, Jinan 250022, China; Jinan Key Laboratory of Pediatric Respiratory Diseases, Jinan Children’s Hospital, Jinan 250022, China

**Keywords:** hepatoblastoma, microRNAs, molecular mechanisms, diagnosis, treatment, precision medicine

## Abstract

Hepatoblastoma (HB) is the most common malignant liver tumor in children. Early diagnosis and effective treatment are crucial for improving the prognosis of children with HB. In recent years, microRNAs (miRNAs), an important class of noncoding RNA molecules, have been increasingly recognized for their key regulatory roles in the occurrence, development, and treatment of HB. This review systematically reviews the expression characteristics, molecular mechanisms, and potential application value of miRNAs in the diagnosis and treatment of HB. Research indicates that the interaction network between miRNAs and long noncoding RNAs and circular RNAs has a significant effect on the development of HBs. miRNAs regulate signaling pathways, such as the Wnt/β-catenin, mitogen-activated protein kinase, phosphatidylinositol 3-kinase/protein kinase B, and Janus kinase 2/signal transducer and activator of transcription 3 pathways, and also play critical roles in the biological behavior of HBs. Furthermore, the progress of preclinical research on miRNAs as biomarkers and therapeutic targets provides new ideas and directions for precision medicine in HB. Finally, this article looks forward to the future development directions of miRNAs in precision medicine for HBs, emphasizing their important potential in improving diagnostic accuracy and treatment efficacy.

## Introduction

Hepatoblastoma (HB), the most common malignant liver tumor in children, originates from undifferentiated hepatic progenitor cells during embryonic development [1]. HB primarily occurs in children <5 years of age [2]. Although it is rare globally, HB accounts for a significant proportion of pediatric liver cancer cases, with lower survival rates in high-risk and metastatic cases [3]. The pathogenesis of HB is complex and highly heterogeneous [4].

In recent years, studies have shown that microRNAs (miRNAs), important noncoding RNAs, play crucial regulatory roles in the occurrence and development of HB [5]. miRNAs are a class of small molecules (19–25 nucleotides), single-stranded, and noncoding RNAs that regulate gene expression by specifically binding to target gene mRNAs [6], thereby affecting the biological behavior of tumor cells, including their proliferation, migration, invasion, angiogenesis, and drug resistance [7]. Recent research has shown that miRNAs form complex regulatory networks with long noncoding RNAs (lncRNAs), circular RNAs (circRNAs), and other noncoding RNAs through the competitive endogenous RNA (ceRNA) mechanism, which has significant biological implications for the occurrence and progression of HB [8]. miR-122 is a liver-specific miRNA that has been shown to play an important role in HB by regulating the proliferation and cycle of hepatocytes, thus influencing tumor development [9]. Additionally, the dysregulation of miRNA expression is closely related to the clinical features and prognosis of HB, providing new potential targets for its early diagnosis and treatment [10].

In the microenvironment of HBs, miRNAs promote the invasive ability of tumor cells by regulating intercellular interactions and signaling pathways. Studies have shown that miR-21 can inhibit the apoptosis of hepatoblasts, thereby promoting tumor progression [11]. Moreover, lncRNAs such as ZEB1-AS1 have been shown to regulate the expression of their target genes by adsorbing miRNAs, thus affecting the occurrence and development of hepatocellular carcinoma (HCC) [12, 13]. Treatment strategies for HB increasingly emphasize the clinical application potential of miRNAs. The use of specific miRNA inhibitors or agonists for treatment may provide new therapeutic options for HB patients. Furthermore, the construction of ceRNA networks offers new perspectives for the functional study of miRNAs [14], helping to elucidate their mechanisms of action in HB. Through in-depth research on these lncRNAs, RNA-based targeted therapies are expected to be developed in the future, thereby improving the prognosis of HB patients. miRNAs play important roles in the occurrence, development, and clinical application of HB, and future research should further reveal their molecular mechanisms and explore their clinical application potential in HB treatment.

## Key role of miRNAs in HB

### Expression characteristics and functions of miRNAs in HB

The pathogenesis of HB is complex and involves multiple molecular pathways and regulatory factors. In recent years, an increasing number of studies have shown that miRNAs play important roles in the development of HB. miRNAs can inhibit the expression of target mRNAs by binding to them, thereby regulating biological processes such as cell proliferation, differentiation, metastasis, and apoptosis [15]. Research has shown that specific miRNAs exhibit abnormal expression patterns in HBs. One study revealed that miR-203 is significantly downregulated in HB tissues, whereas the expression of the lncRNA CRNDE is upregulated, and this expression imbalance is closely related to the size and stage of the tumor. Functional experiments indicate that the overexpression of miR-203 can inhibit the proliferation, migration, and angiogenesis of HB cells, suggesting that miR-203 may play a role in suppressing tumor development in HB [16]. Additionally, the lncRNA MIR210HG is highly expressed in HB tissues and cell lines, and it regulates the expression of forkhead box O 6 (FOXO6) by binding to miR-608, thereby affecting the cell migration and invasion capabilities of HBs. Inhibiting the expression of MIR210HG can significantly reduce the proliferation and migration abilities of HB cells, providing new insights for the treatment of HB [17].

Research has also revealed that the lncRNA SNHG9 regulates the Wnt3a pathway through miR-23a-5p, promoting tumorigenesis in HB. This observation indicates that lncRNAs not only play important roles in the occurrence of HB but may also influence the activity of cancer-related signaling pathways by regulating miRNAs [18]. These findings suggest that the interaction between miRNAs and lncRNAs may be a key regulatory mechanism in the occurrence and development of HB. In another study, the lncRNA TUG1 was shown to be upregulated in HB cells and to activate the Janus kinase 2/signal transducer and activator of transcription (JAK2/STAT3) signaling pathway by inhibiting miR-204–5p, thereby promoting angiogenesis [19]. These results further emphasize the importance of lncRNAs in the tumor microenvironment of HBs, especially in regulating tumor angiogenesis and tumor progression.

In summary, the study of the expression characteristics and functions of miRNAs and lncRNAs in HB provides important clues for understanding the molecular mechanisms of this disease. miRNAs not only play significant roles in the development of HB but may also serve as potential biomarkers and therapeutic targets, offering new options for future clinical interventions. Researchers should continue to explore the specific mechanisms of these miRNAs to provide more effective strategies for the early diagnosis and treatment of HB.

#### Heterogeneity in miRNA expression profiles

In the study of HB, transcriptome analysis revealed significant changes in the expression profiles of miRNAs. Multiple studies have shown that the expression of certain miRNAs is significantly upregulated in HB tissues whereas the expression of other miRNAs is significantly downregulated. These abnormally expressed miRNAs are closely related to the biological behavior of HB and are involved in regulating key biological processes such as tumor cell proliferation, migration, angiogenesis, and metabolism. Specifically, upregulated miRNAs are associated with the malignant phenotype of HB and can promote the proliferation and migration of tumor cells. A recent study utilized bioinformatics methods to mine potential differentially expressed genes closely related to HB in the gene expression dataset GSE131329 and analysed the targeted interactions with miRNAs through miRTarBase. A total of 594 differentially expressed miRNAs were identified, including 221 upregulated and 373 downregulated genes, that are involved in various cellular and metabolic processes and human diseases, including tumors. For the first time, miR-193b and miR-760 were found to be closely related to HB [2]. These findings may help to reveal the potential mechanisms of HB and provide new insights for better prognosis and treatment strategies.

Recent studies have shown that miR-214 promotes the proliferation and survival of HB cells by regulating signaling pathways related to the cell cycle and apoptosis [20]. Moreover, miR-492 is believed to play an important role in regulating cellular metabolism and proliferation, with its upregulation associated with the progression of HB [21]. On the other hand, the downregulation of miR-4510 is also significantly associated with the malignant characteristics of HB. Research has shown that miR-4510 inhibits the transcriptional activity of β-catenin without affecting its expression, reducing the viability of HuH6 cells [22]. Additionally, miR-186, another downregulated miRNA, significantly affects the migration, invasion, and proliferation of liver tumor cells [23]. In summary, the changes in miRNA expression profiles not only provide profound insights into the biological characteristics of HB but also lay the foundation for potential therapeutic targets. Through the regulatory mechanisms of these miRNAs, new treatment strategies may be developed in the future to improve the prognosis of HB patients.

#### Regulation of tumor cell biological behavior by miRNAs

**Figure 1.**
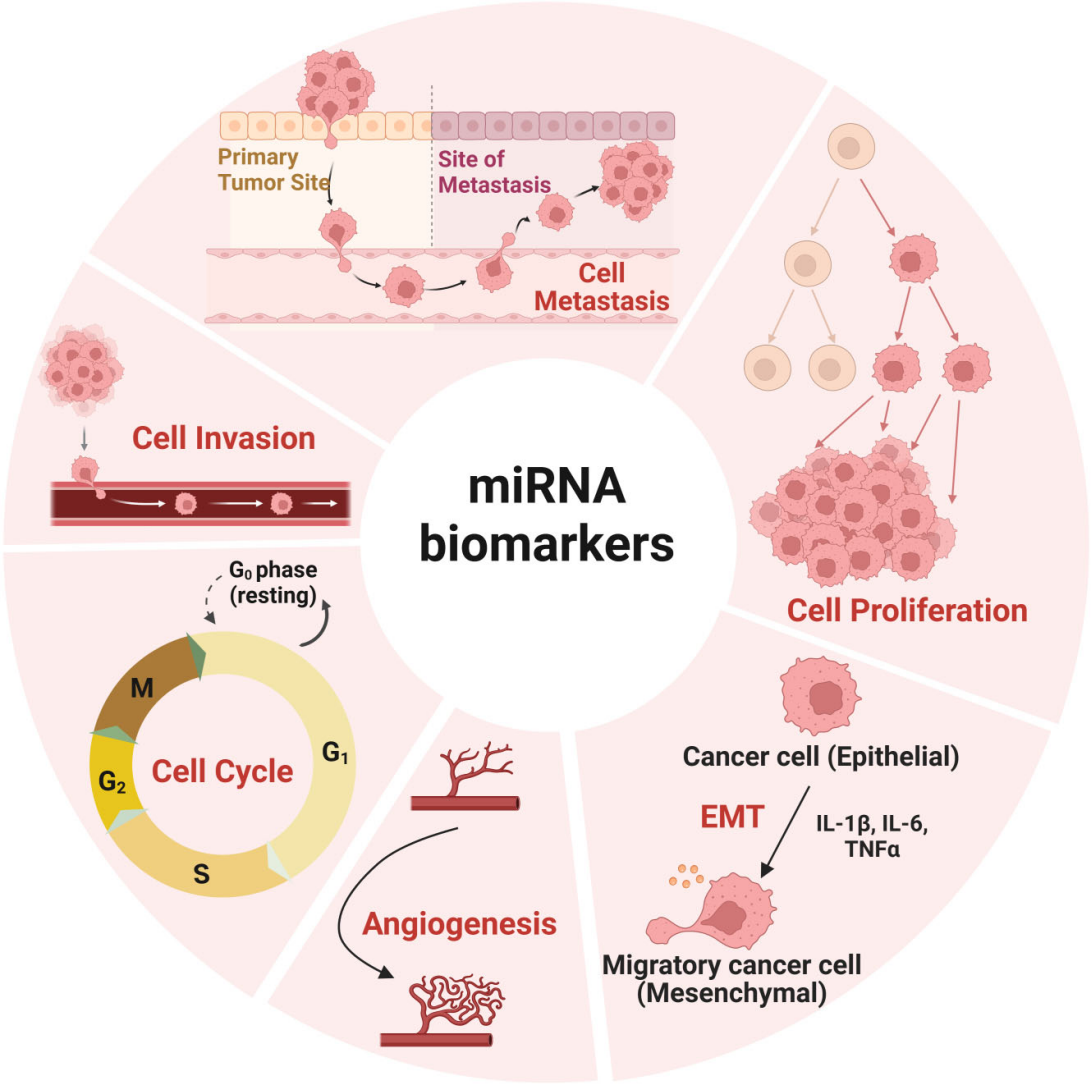
miRNAs play a central regulatory role in the occurrence and development of HB. The mechanism of action relies mainly on the ability of miRNAs to specifically recognize and bind to the 3' untranslated region of target gene mRNAs, achieving fine-tuned regulation of gene expression at the posttranscriptional level. This regulation profoundly affects various key biological processes in HB tumor cells, including but not limited to abnormal proliferation and uncontrolled growth, dysregulation of the cell cycle process, enhanced invasive and migratory capabilities, acquisition of distant metastatic potential, activation of angiogenesis, and induction of epithelial‒mesenchymal transition (EMT). Notably, individual miRNAs often exhibit significant pleiotropy; they may simultaneously act on multiple target genes or signaling pathways, thereby producing additive or synergistic effects on one or even multiple biological processes mentioned above. Through this complex and extensive regulatory network, specific miRNAs (or miRNA combinations) can powerfully drive the malignant phenotypic evolution of HB, ultimately promoting tumor progression, metastasis, and treatment resistance.

**Figure 2.**
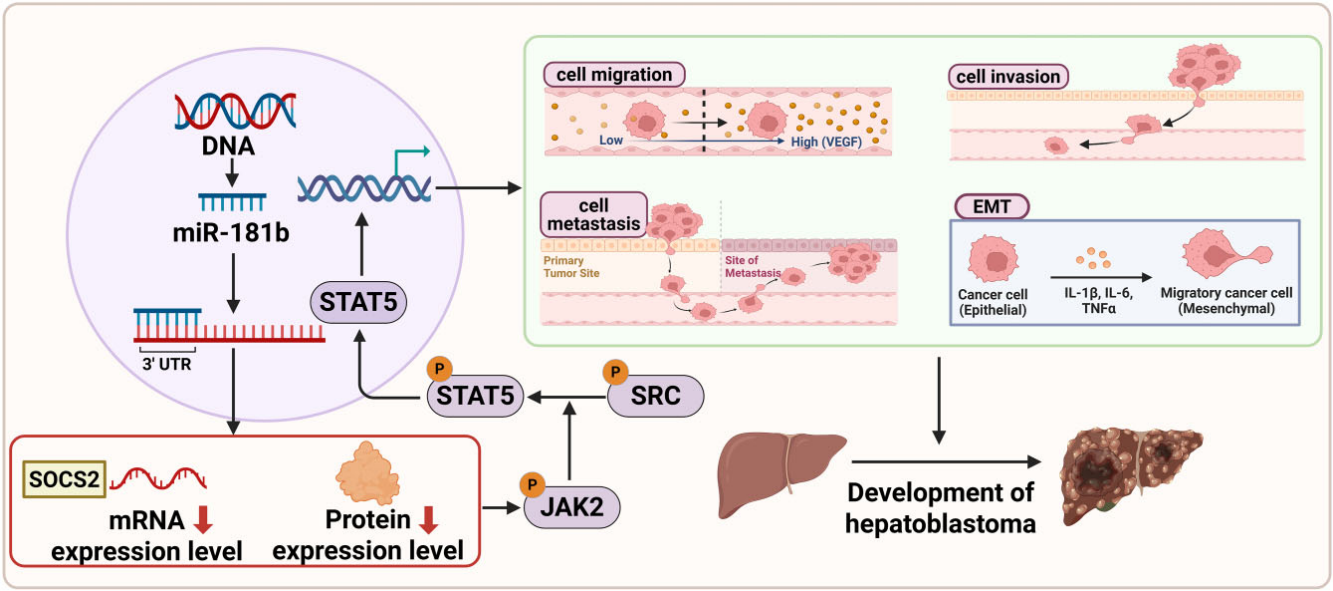
The miR-181b/SOCS2/JAK2/STAT5 signaling axis promotes the progression of HB. miR-181b-5p is highly expressed in HB tissues and cells. In addition, SOCS2 is a direct target gene of miR-181b-5p, which can specifically target and bind to the 3' untranslated region of SOCS2 mRNA. The binding of these two proteins leads to significant negative regulation of SOCS2 at both the mRNA and protein levels, indicating that the expression of SOCS2 is effectively suppressed by miR-181b-5p. By targeting and inhibiting the expression of SOCS2, the negative regulatory effect of SOCS2 on the JAK2/STAT5 pathway is relieved, resulting in abnormal activation of this pathway, which enhances HB cell migration, invasion, metastasis, and epithelial‒mesenchymal transition, ultimately promoting the progression of HB. Therefore, the miR-181b-5p/SOCS2/JAK2/STAT5 signaling axis has been identified as a key oncogenic pathway driving the occurrence and development of HB, providing important evidence for understanding the molecular basis of HB and developing potential targeted therapeutic strategies.

The regulatory role of miRNAs in HB has become a key area of research in tumor biology in recent years (Fig. 1, Table 1). Our team recently reported that miR-181b, a tumor-promoting factor, is highly expressed in HB tissues and HB cell lines. The overexpression of miR-181b enhances the migration and invasion capabilities of HB cells and increases the potential for lung metastasis *in vivo*. Dual-luciferase reporter gene assays confirmed that suppressor of cytokine signaling 2 (SOCS2) is a direct target of miR-181b and that the miR-181b-5p/SOCS2/JAK2/STAT5 signaling axis promotes the metastasis of HB (Fig. 2). The signaling axis may be a therapeutic target for HB [24]. Some studies have shown that miR-135a can affect the growth cycle of HB tumor cells, thereby inhibiting the activation of the Notch pathway and ultimately suppressing the proliferation of HB cells [25]. Wang *et al*. reported that the expression of miR-489 is significantly reduced in HCC tissues and can significantly inhibit the occurrence and development of HCC cells, possibly by downregulating the expression of SRY-related high mobility group box 4 (SOX4) to suppress cell proliferation. Further studies indicated that the SOX4 gene in tumor cells can serve as a regulatory factor for miR-489 in HCC [26].

**Table 1.** Regulation of tumor cell biological behavior by miRNAs.

miRNA biomarkers	Biological behavior of tumor cells
miR-22, miR-122, miR-492, miR-203, miR-206, miR-126, miR-186, miR-214 miR-514a-5p, miR-23a-5p, miR-7–5p, miR-204–5p, miR-154–3p, miR-6838–5p, miR-329–3p, miR-378a	Cell proliferation
miR-203, miR-608, miR-186, miR-181b, miR-205–5p, miR-514a-5p, miR-329–3p, miR-492, miR-354–3p	Cell metastasis
miR-608, miR-186, miR-181b, miR-205–5p, miR-514a-5p, miR-378a	Cell invasion
miR-214, miR-122, miR-135a	Cell cycle
miR-203, miR-204–5p	Angiogenesis
miR-1246, miR-181b	Epithelial‒mesenchymal transition

Moreover, miR-23a-5p promotes the occurrence of HB by regulating the Wnt3a signaling pathway. Research has shown that the upregulation of miR-23a-5p is closely related to the overexpression of SNHG9 and that high expression of SNHG9 promotes the proliferation and clonogenic ability of HB cells. By directly interacting with Wnt3a, miR-23a-5p enhances tumor incidence in liver cells [7, 18]. Therefore, the regulation of miR-23a-5p may be a new target for the treatment of HB. Additionally, miR-204–5p plays an important role in HB. It regulates angiogenesis by inhibiting the JAK2/STAT3 signaling pathway. Studies have shown that the expression of miR-204–5p is significantly decreased in HB tissues and cells and that its inhibitory effect can prevent the occurrence of angiogenesis, thereby affecting tumor growth and metastasis [19]. Therefore, the regulation of miR-204–5p may provide new therapeutic strategies for HB. In summary, miRNAs regulate the biological behavior of HB cells through various mechanisms, including cell proliferation, migration, and angiogenesis. These findings provide an important basis for understanding the molecular mechanisms of HB and open new directions for future treatment strategies.

#### miRNA and tumor stem cell characteristics

The occurrence of HB is closely related to changes in tumor stem cell characteristics. Studies have shown that the circRNA CDR1as promotes tumor stem cell characteristics and tumor progression by sponging miR-7–5p to regulate the expression of Kruppel-like factor 4 (KLF4). Specifically, CDR1as acts as a competitive ceRNA, reducing the inhibitory effect of miR-7–5p on KLF4 by binding to and inhibiting miR-7–5p, leading to the upregulation of KLF4 expression [27]. KLF4 is a transcription factor closely associated with tumor stem cell characteristics and is involved in cell proliferation, differentiation, and self-renewal [28]. In HB cell lines, high expression of CDR1as is associated with enhanced tumor stem cell characteristics. Through flow cytometry and sphere-formation assays, Chen *et al*. reported that the knockdown of CDR1as led to a significant decrease in the proportion of tumor stem cells in HB cell lines, indicating its important role in maintaining tumor stem cell characteristics. Furthermore, the knockdown of CDR1as inhibited cell proliferation and colony formation, further supporting its role as a tumor-promoting factor. Research has shown that the expression level of miR-7–5p is negatively correlated with that of KLF4 and that the upregulation of miR-7–5p can inhibit KLF4 expression, thereby suppressing tumor stem cell characteristics. Therefore, the presence of CDR1as maintains KLF4 expression, enhancing the tumor stem cell characteristics of HB [29]. Additionally, studies surrounding CDR1as have revealed its potential mechanisms in other cancers. In various types of cancers, circRNAs influence the biological behavior of tumor cells by regulating miRNA expression, demonstrating their potential as tumor biomarkers and therapeutic targets.

Hu *et al*. reported that miR-126 is upregulated in HB, and exosomes derived from HB cells significantly increased the proportion of CD44+ CD90+ CD133+ cells, as well as the expression of interleukin 6 (IL-6), Oct4, SRY, and transforming growth factor-β (TGF-β) in bone marrow mesenchymal stem cells (BMSCs). In contrast, exosomes with downregulated miR-126 reversed these phenomena. The downregulation of miR-126 significantly inhibited tumor growth in HBs and reduced the proportion of CD44+ CD90+ CD133+ cells, increasing the expression of IL-6, Oct4, SRY, TGF-β, and β-catenin in mouse tumor tissues. Moreover, exosomes with downregulated miR-126 inhibited the differentiation of BMSCs into cancer stem cells, suggesting that exosomal miR-126 from HB cells promotes the occurrence of HB by inducing mesenchymal stem cells to differentiate into cancer stem cells [30]. In summary, circRNAs play a role in promoting tumor stem cell characteristics in HB by regulating miRNA expression, providing new research directions and potential therapeutic strategies.

In summary, miRNAs play pivotal roles in the pathogenesis of HB by regulating key oncogenic signaling pathways and cellular processes. Key miRNAs such as miR-203, miR-204–5p, and miR-4510 are frequently downregulated in HB and function as tumor suppressors by inhibiting proliferation, migration, angiogenesis, and stemness. In contrast, miR-181b, miR-23a-5p, miR-214, and miR-492 are often upregulated and promote tumor progression. These miRNAs exert their effects through critical pathways, including the JAK2/STAT3, Wnt/β-catenin, Notch, and SOCS2/JAK2/STAT5 signaling pathways. Additionally, noncoding RNAs such as lncRNAs (CRNDE, MIR210HG, SNHG9, TUG1) and circRNAs (CDR1) interact with miRNAs (miR-7–5p, miR-608) to modulate the expression of downstream targets such as KLF4 and FOXO6, further influencing tumor stemness and malignancy. The dysregulation of miRNA expression profiles highlights their potential as biomarkers and therapeutic targets, offering novel insights for HB diagnosis and treatment.

### miRNA and lncRNA regulatory axes

In the study of HB, the role of lncRNAs as regulatory molecules has received increasing attention, especially with respect to their impact on key biological signaling pathways through the regulation of miRNAs, particularly in the occurrence and development of tumors. lncRNAs play important roles in biological processes such as cell proliferation, migration, and invasion, and they influence the progression of HB by regulating specific signaling pathways. NBR2, which is a lncRNA, also plays a significant role in the tumorigenesis of HB. Research shows that NBR2 exacerbates the malignancy of HB cells by competing with T-cell factor-7 (TCF7) for miR-22 binding, thereby alleviating the inhibitory effect of miR-22 on TCF7. The lncRNA NBR2 may be a promising target for inhibiting HB cell proliferation under glucose starvation conditions [31]. LINC01023 is also a noteworthy lncRNA that promotes the progression of HB by regulating the interaction between miR-378a-5p and Wnt3. The upregulation of LINC01023 is significantly associated with the proliferation and colony formation of HB cells, whereas its downregulation leads to increased apoptosis, indicating that LINC01023 promotes the occurrence of HB [32].

Zhang *et al*. reported that MIR205HG, a lncRNA, activates the mitogen-activated protein kinase (MAPK) and phosphatidylinositol 3-kinase/protein kinase B (PI3K/AKT) signaling pathways by regulating the expression of miR-514a-5p and miR-205–5p [33]. These two miRNAs play important roles in the progression of HB. First, MIR205HG was found to be significantly overexpressed in HB tissues and cell lines, suggesting that it may act as a tumor promoter. Studies have shown that the inhibition of MIR205HG can significantly reduce the proliferation, migration, and invasion capabilities of HB cells, thereby slowing tumor growth. MIR205HG effectively relieves the inhibitory effects of these miRNAs on their target genes by competitively binding to miR-514a-5p and miR-205–5p, promoting the activation of the MAPK and PI3K/AKT signaling pathways, both of which are closely related to cell proliferation, survival, and migration. The MAPK signaling pathway plays a key role in cellular responses, proliferation, and survival, and its activation is often associated with the malignant characteristics of tumor cells. In HB, MIR205HG activates MAPK9 by regulating miR-514a-5p, further promoting the proliferation and migration of HB cells. Moreover, the PI3K/AKT signaling pathway plays an important role in the survival and proliferation of cancer cells. The activation of the MAPK and PI3K/AKT signaling pathways by MIR205HG not only supports the biological behavior of HB but also provides new targets for the development of future therapeutic strategies. Further research on the interactions between MIR205HG and other lncRNAs, miRNAs, and their target genes may reveal more complex regulatory networks, providing a deeper perspective for understanding the molecular mechanisms of HB.

In addition, TUG1, another lncRNA, also plays an important role in the angiogenesis of HB. Studies have shown that TUG1 promotes angiogenesis in HB cells by inhibiting miR-204–5p and activating the JAK2/STAT3 signaling pathway. These findings provide a new potential target for the treatment of HB [19]. Additionally, research has shown that SNHG1, as a lncRNA, promotes the proliferation, migration, and invasion of HB cells by regulating the miR-6838–5p/PIM3/RhoA axis. These data may provide new therapeutic targets for HB treatment [34].

In summary, the regulatory axis between miRNAs and lncRNAs plays a key role in the biological characteristics of HB. These findings not only provide an in-depth understanding of the biological mechanisms of HB but also offer new perspectives and potential targets for future therapeutic strategies. Further research on the mechanisms of action of these lncRNAs may lead to new breakthroughs in the clinical treatment of HB.

### Interactions between miRNAs and circRNAs

In the study of HB, circRNA, an important lncRNA, has gradually been recognized for its key role in tumor biology. circRNAs affect cell proliferation, migration, stemness maintenance, and tumor microenvironment regulation by forming competitive ceRNA networks with miRNAs [35], thus playing important roles in the occurrence and development of tumors. Specifically, the circRNA CDR1 is highly expressed in HB cells and inhibits the expression of the stem cell maintenance factor KLF4 by acting as a sponge for miR-7–5p, thereby promoting the proliferation and maintenance of the stemness of tumor cells. This mechanism indicates that circRNAs not only are products of RNA transcription but also play a direct regulatory role in the biological functions of tumor cells [29]. Research has shown that circ-STAT3 acts as a sponge for miR-29a/b/c-3p, with Gli2 acting as a transcription factor for circ-STAT3, increasing the expression of STAT3 and Gli2. circ-STAT3 promotes the proliferation, invasion, migration, stemness, and tumor growth of HB cells by upregulating STAT3 and Gli2, suggesting that circ-STAT3 may be a biomarker for HB [36].

circ-CCT2 is a novel circular RNA derived from the CCT2 gene. A recent study by Zhu *et al*. revealed that circ-CCT2 stabilizes polypyrimidine tract-binding protein 1 (PTBP1) mRNA and activates the Wnt/β-catenin signaling pathway by recruiting and upregulating the TAF15 protein, thereby promoting the progression of HB and suggesting potential therapeutic targets [37]. Cui *et al*. reported that the upregulation of O-glycosylated La-related protein 1 (LARP1) mediated by circCLNS1A promotes the occurrence and progression of HB through the la-related protein 1 (LARP1)/dickkopf4 (DKK4)/β-catenin axis, with LARP1 and DKK4 being promising therapeutic targets and diagnostic biomarkers for HB [38]. The interaction between circRNAs and miRNAs plays a crucial role in the biology of HB. Studies have shown that the dynamic balance of these lncRNAs not only affects the biological characteristics of HB but also may become key to future targeted therapies. Future in-depth exploration of the mechanisms of circRNAs in HB is highly important for revealing their biological characteristics and finding new therapeutic targets while also providing new ideas and strategies for the clinical treatment of HB.

In HB, circRNAs function as pivotal regulatory molecules by acting as ceRNAs for miRNAs, thereby governing key oncogenic processes, notably: CDR1 promotes proliferation and stemness by sponging miR-7–5p to inhibit KLF4; circSTAT3 enhances tumor growth and invasion by sequestering miR-29a/b/c-3p to upregulate STAT3 and Gli2; circCCT2 drives progression by recruiting TAF15 to stabilize PTBP1 and activate Wnt/β-catenin signaling; and circCLNS1A facilitates tumorigenesis via the LARP1/DKK4/β-catenin axis, collectively establishing these circRNAs as promising diagnostic biomarkers and therapeutic targets in HB.

### Key signaling pathways regulated by miRNAs

**Figure 3.**
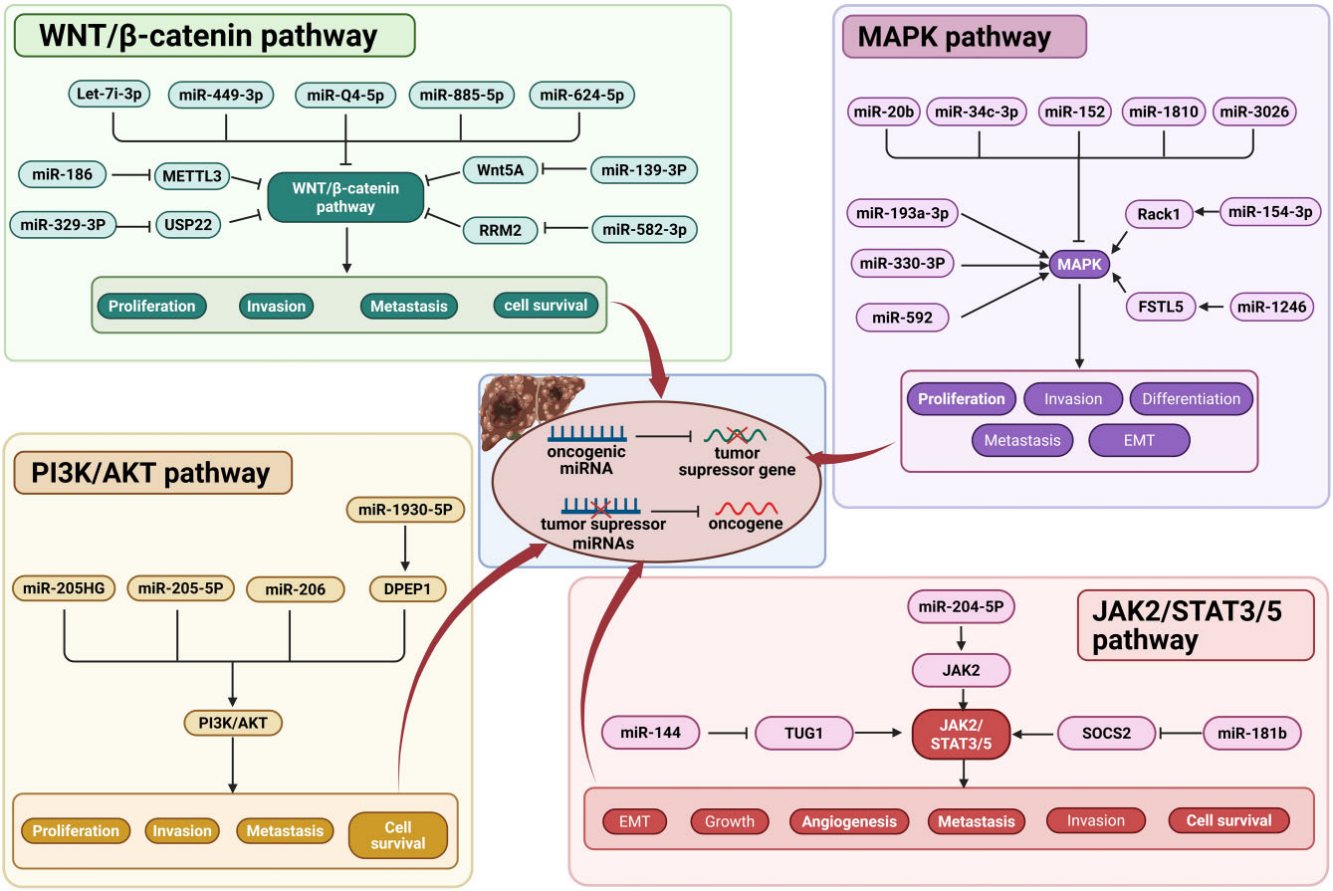
miRNA-regulated signaling in HB progression. miRNAs have been confirmed as critical regulatory factors in the occurrence and progression of HB. In HB, various types of dysregulated miRNA expression, including significant upregulation or downregulation, are observed. These dysregulated miRNAs profoundly impact multiple core signaling pathways that drive tumor development through posttranscriptional regulatory mechanisms (mainly by binding and inhibiting or degrading their target mRNAs). Among them, the Wnt/β-catenin pathway (which regulates cell survival, proliferation, differentiation, and stem cell characteristics), the MAPK pathway (which mediates cell proliferation, differentiation, survival, and migration), the PI3K/AKT pathway (which promotes cell survival, growth, metabolism, invasion, and metastasis), and the JAK2/STAT3 pathway (which is involved in cell survival, immune evasion, invasion, metastasis, and angiogenesis) are the key pathways most significantly regulated by miRNAs. Specifically, dysregulated miRNAs disrupt pathway activity by precisely targeting the mRNAs of key genes in these pathways (such as oncogenes or tumor suppressor genes). This molecular-level disturbance is directly related to a series of malignant biological processes in HB, including tumor initiation, sustained growth and development, local tissue invasion, and distant metastatic spread.

miRNAs play critical regulatory roles in the development of HB by influencing tumor cell proliferation, migration, and invasion through multiple signaling pathways. Recent studies in this field have revealed several key pathways, including the Wnt/β-catenin, MAPK, PI3K/AKT, and JAK2/STAT3 signaling pathways (Fig. 3).

#### Wnt/β-catenin signaling pathway

The Wnt/β-catenin signaling pathway plays a role in various physiological processes, including differentiation, proliferation, apoptosis, migration, invasion, and tissue homeostasis. It is particularly important in the progression of HB. Studies have shown that miRNAs regulate the proliferation, invasion, metastasis, and drug sensitivity of tumor cells by modulating key regulatory factors in the classical Wnt/β-catenin signaling pathway [39, 40]. The Wnt/β-catenin pathway plays a critical role in the occurrence and progression of HB. Since Wnt/β-catenin signaling is a major therapeutic target for HB, some studies have explored the role of miRNAs in this pathway. For example, Indersie *et al*. reported that four miRNAs (let-7i-3p, miR-449–3p, miR-624–5p, and miR-885–5p) were reduced in HB samples compared with normal liver controls. They also revealed that these miRNAs regulated the expression of β-catenin and inhibited cell proliferation and Wnt/β-catenin activity *in vitro*. Additionally, miR-624–5p inhibits tumor growth *in vivo* and directly targets β-catenin through its 3' untranslated region [41]. Another study indicated that transfection with the miR-4510 precursor led to reduced cell proliferation and may induce apoptosis in HuH6 cells by inhibiting the Wnt/β-catenin pathway, suggesting the potential utility of this miRNA as a therapeutic target [22].

Moreover, research has shown that miR-186 regulates the proliferation and invasion capabilities of tumor cells by inhibiting N6-methyladenosine (m6A) methyltransferase-like 3 (METTL3), thereby affecting the Wnt/β-catenin signaling pathway. Specifically, the upregulation of METTL3 is associated with the malignant characteristics of HB, promoting the proliferation and migration of HB cells by regulating the expression of the Wnt/β-catenin signaling pathway [42]. In HB cells, the expression of miR-186 is significantly reduced, leading to the upregulation of METTL3, which in turn activates the Wnt/β-catenin signaling pathway. The key mechanism involved is the direct inhibitory effect of miR-186 on METTL3, and the upregulation of miR-186 can significantly inhibit the proliferation and invasion capabilities of HB cells. These findings provide new biomarkers and targets for the treatment of HB. Additionally, miR-186 influences cell growth and survival by regulating downstream signaling molecules. Therefore, the interaction of the miR-186/METTL3/Wnt signaling pathway plays an important role not only in the occurrence and development of HB but also in tumor prognosis and treatment outcomes [43]. Wu *et al*. reported that the miR-139–3p/Wnt5A signaling axis inhibited the metastasis of HB, indicating that miR-139–3p and Wnt5A may be potential targets for HB treatment [44]. Xin *et al*. discovered that miR-329–3p could inhibit the proliferation and migration of HepG2 cells by suppressing the ubiquitin-specific peptidase 22 (USP22)-Wnt/β-catenin pathway, providing new insights into the etiology and potential treatment of HCC [45]. Xu *et al*. reported that miR-582–3p targets ribonucleotide reductase regulatory subunit M2 (RRM2) and inhibits the tumorigenesis of HCC by regulating the Wnt/β-catenin signaling pathway [46]. Moreover, Bhandari *et al*. demonstrated that LINC01023 contributes to hepatoblastoma development via the LINC01023/miR-378a-5p/Wnt3 axis, suggesting that it is a promising therapeutic target and prognostic biomarker [32].

In summary, miRNAs regulate the proliferation and invasion of HB cells by affecting the Wnt/β-catenin signaling pathway, revealing their value as potential biomarkers and therapeutic targets. These findings not only provide new perspectives for understanding the molecular mechanisms of HB but also open new avenues for the clinical treatment of HB.

#### MAPK signaling pathway

MAPK signal transduction, a highly conserved signaling pathway, plays an important role in various biological processes, such as cell growth, proliferation, differentiation, and stress responses. miRNAs interact with the MAPK signaling pathway in cancer progression, and MAPK signaling is often overexpressed during cancer progression. These findings indicate that miRNAs play a key role in the development of human cancers in conjunction with this signaling pathway. Some miRNAs, such as miR-20b, miR-34c-3p, miR-152, miR-181a, and miR-302b, regulate tumorigenesis by inhibiting the MAPK signaling pathway, whereas miR-193a-3p, miR-330–3p, and miR-592 activate this signaling pathway [47]. In studies on HB, the lncRNA Linc00205 was shown to activate the MAPK pathway by regulating the miR-154–3p/ROCK1 axis, thereby promoting the proliferation and migration of tumor cells [48]. Specifically, Linc00205 binds to miR-154–3p, inhibiting its suppressive effect on its target gene ROCK1 and thus upregulating the expression of ROCK1. This effect not only promotes the proliferation and migration of HB cells but also facilitates the malignant progression of tumors by activating the MAPK signaling pathway. In HB, activation of the MAPK pathway typically leads to increased cell proliferation and migration, which is closely related to the clinical prognosis of HB patients. Through in-depth studies of the Linc00205/miR-154–3p/ROCK1/MAPK axis, we hope to identify new targeted therapeutic strategies to intervene in the progression and metastasis of HB.

Chen *et al*. reported that the level of miR-1246 in HCC tissues was significantly greater than that in adjacent tissues and that the enrichment of miR-1246 in exosomes derived from HepG2 cells was greater than that in those derived from HepG2 cells. Exosomes derived from HCC cells significantly enhanced the invasion, migration, proliferation, and epithelial‒mesenchymal transition (EMT) of HCC cells, whereas exosomes loaded with miR-1246 inhibitors suppressed these biological functions. Further mechanistic studies indicated that miR-1246 promotes the progression of HCC through FSTL5 and the extracellular signal-regulated kinase (ERK)/p38 MAPK pathway *in vitro* [49]. These findings highlight the importance of miRNAs as tumor biomarkers and therapeutic targets, especially in pediatric malignancies such as HB, where exploring their mechanisms of action will help improve patient prognosis and survival rates. In summary, Linc00205 activates the MAPK signaling pathway by regulating the miR-154–3p/ROCK1 axis, promoting the proliferation and migration of HB cells. These findings provide new perspectives for understanding the pathogenesis of HB and lay the foundation for the development of future therapeutic strategies.

#### PI3K/AKT signaling pathway

The PI3K/AKT signaling pathway is widely regarded as a core regulatory factor in cell growth, proliferation, metabolism, angiogenesis, and metastasis [50, 51]. The PI3K/AKT pathway is activated by receptor tyrosine kinases that can be stimulated by cytokines or epidermal growth factors. The activation of this pathway promotes the activation of many intracellular proteins through phosphorylation [52]. Studies have confirmed that the PI3K/AKT signaling pathway plays a key role in the occurrence and development of HB [53]. miRNAs play crucial roles as upstream or downstream targets in the PI3K/AKT pathway, and abnormal activation of this pathway contributes to cancer development. Increasing evidence suggests that miRNAs can control the PI3K/AKT pathway, thereby regulating intracellular biological processes. Gene expression related to cancer can be regulated through the miRNA/PI3K/AKT axis, thus controlling cancer progression [54]. Recent studies have shown that the lncRNA MIR205HG activates the PI3K/AKT signaling pathway by competitively binding to miR-205–5p, thereby promoting tumor progression. High expression of MIR205HG has been widely observed in HB tissues and cell lines, providing evidence for its potential as a tumor marker and therapeutic target [33]. MIR205HG is not only closely related to the growth of HBs but also regulates cell proliferation, migration, and invasion capabilities by affecting various signaling pathways, especially the PI3K/AKT pathway. Research indicates that MIR205HG can competitively bind to miR-205–5p, thereby alleviating the inhibition of downstream target genes, activating the PI3K/AKT signaling pathway, and promoting a malignant phenotype in cells.

Moreover, activation of the PI3K/AKT signaling pathway is closely related to the prognosis of HB patients. Studies have shown that abnormal activation of the PI3K/AKT signaling pathway can lead to uncontrolled cell proliferation and increased antiapoptotic capabilities. Chen *et al*. reported that miR-206 promotes HB growth by enhancing the PI3K/Akt/mammalian target of rapamycin (mTOR) signaling pathway [55]. Therefore, interventions targeting this pathway may provide new strategies for the treatment of HB. Notably, other studies have indicated that the PI3K/AKT signaling pathway may play different roles in the various biological characteristics of HB [56]. Cui *et al*. reported that miR-193a-5p can regulate the tumorigenesis and progression of HB through the PI3K/Akt/mTOR signaling pathway. The miR-193a-5p/dipeptidase 1 (DPEP1) axis can serve as an effective therapeutic and prognostic biomarker for HB patients [57]. Xu *et al*. reported that downregulating the expression of miRNA-196b inhibits the proliferation, migration, and invasion of HepG2 cells while promoting their apoptosis via the PI3K/AKT signaling pathway [58]. These findings further emphasize the importance of miRNAs in the development of HB through the regulation of the PI3K/AKT signaling pathway. In summary, miRNAs promote the progression of HB by activating the PI3K/AKT signaling pathway, providing important clues for understanding the molecular mechanisms of HB and its treatment. Future research can explore how to effectively intervene in this pathway to improve the prognosis of HB patients.

#### JAK2/STAT3 signaling pathway

As one of the important signaling pathways in cells, the JAK2/STAT3 pathway plays a key role in cell proliferation, differentiation, survival, and angiogenesis by affecting the activation state of downstream effector molecules. The activation of the JAK2/STAT3 signaling pathway is associated with the occurrence and development of tumors. It contributes to the formation of the inflammatory tumor microenvironment and is closely related to the occurrence and development of many human tumors [59]. In a study of HB, the lncRNA TUG1 was found to activate the JAK2/STAT3 signaling pathway by inhibiting miR-204–5p, thereby promoting angiogenesis in HB [19]. The development and prognosis of HB are often influenced by various genetic and epigenetic factors. The upregulation of TUG1 is common in HB tissues and cells, indicating its potentially important role in tumor occurrence and development. Research has shown that TUG1 inhibits the negative regulatory effect of miR-204–5p on JAK2 by binding to it. JAK2 is an important cellular signaling molecule, and its activation can trigger a cascade of downstream signaling pathways, including the phosphorylation of STAT3. STAT3 is a transcription factor that can promote various biological processes, such as cell survival, proliferation, and angiogenesis [60]. In the microenvironment of HB, the activation of the JAK2/STAT3 pathway promotes tumor angiogenesis by increasing the expression of angiogenic factors such as vascular endothelial growth factor (VEGF), which is crucial for tumor growth and metastasis. Lv *et al*. reported that miR-144 can interact with TUG1, promoting the proliferation, migration, and tumorigenesis of HCC by activating the JAK2/STAT3 pathway [61].

Increasing evidence suggests that dysregulation of the JAK/STAT pathway is associated with the proliferation, migration, and invasion of tumor cells [62]. In the liver, the SOCS2/JAK2/STAT5 axis and its negative regulatory effects are crucial for maintaining the normal physiological state of hepatocytes [63]. Previous studies have shown that high-glucose-induced JAK/STAT activation upregulates the expression of miR-181b in glomerular mesangial cells, establishing a link between JAK/STAT signaling and miR-181b for the first time [64]. Our team recently discovered that high expression of miR-181b in HB cells promotes tumor metastasis by inhibiting the expression of SOCS2, leading to the activation of the JAK2/STAT5 signaling pathway and EMT. Conversely, restoring SOCS2 expression or inhibiting JAK2/STAT5 signaling can suppress tumor metastasis in HB. These results indicate that the miR-181b/SOCS2/JAK2/STAT5 signaling axis may be a novel therapeutic target for the prevention and treatment of HB [24, 65]. miRNAs play important roles in the progression of HB by activating the JAK2/STAT3/5 signaling pathway, providing new perspectives for the study of the molecular mechanisms of HB, as well as potential intervention points for future targeted therapies. Researchers can further explore miRNA-related therapies to improve the clinical prognosis of HB patients.

In summary, miRNAs play important roles in the occurrence and development of HB by regulating multiple key signaling pathways. By deeply understanding these signaling pathways and their regulatory mechanisms, researchers can identify new targets and strategies for the early diagnosis and treatment of HB. Future research may reveal more interactions between miRNAs and signaling pathways, laying the foundation for personalized treatment of HB.

#### Integrated miRNA-regulatory networks in HB

In the pathogenesis of HB, a highly integrated molecular regulatory network characterized by functional synergy between key signaling pathways and precise multitarget regulation by miRNAs exists (Table 2). This network utilizes the Wnt/β-catenin and PI3K/AKT pathways as its “backbone”, which forms multilayered positive feedback loops through the shared molecular hub, glycogen synthase kinase-3beta (GSK-3beta). Upon activation of the PI3K/AKT pathway, AKT-mediated phosphorylation of GSK-3β inhibits its kinase activity, thereby preventing the degradation of β-catenin [66, 67]. This mechanism may activate the transcription of Wnt target genes, even in the absence of Wnt ligands or relevant genetic mutations. This synergistic effect is further manifested in the coordinated regulation of downstream target genes; e.g., the expression of key pro-proliferative genes such as c-Myc and cyclin D1 is doubled, while pro-survival signals are concurrently increased, collectively driving cell cycle dysregulation and resistance to apoptosis [68]. From a systems perspective, this “oncogenic alliance” between pathways not only explains the significant heterogeneity observed among tumors harboring similar CTNNB1 mutations but also foreshadows the clinical challenge of acquired resistance to single-pathway targeted therapies.

**Table 2.** Key miRNAs, their targets, regulatory axes, and functional roles in HB.

lncRNA/circRNA	miRNA	Target	Regulatory axis	Functional roles
/	miR-186	METTL3	WNT/β-catenin	Proliferation, invasion, metastasis, cell survival
/	miR-329–3p	USP22	WNT/β-catenin	Proliferation, invasion, metastasis, cell survival
/	miR-139–3p	Wnt5A	WNT/β-catenin	Proliferation, invasion, metastasis, cell survival
/	miR-582–3p	RRM2	WNT/β-catenin	Proliferation, invasion, metastasis, cell survival
SNHG9	miR-23a-5p	Wnt3a	WNT/β-catenin	Proliferation, apoptosis
LINC01023	miR-378a-5p	WNT3	WNT/β-catenin	Proliferation, apoptosis
LINC00205	miR-154–3p	ROCK1	MAPK	Proliferation, invasion, differentiation, metastasis, EMT
/	miR-1246	FSTL5	MAPK	Proliferation, invasion, differentiation, metastasis, EMT
MIR205HG	miR-205–5p	MAPK9	MAPK, PI3K/AKT	Proliferation, migration, invasion
/	miR-206	βKlotho	PI3K/AKT	Growth
/	miR-193a-5p	DPEP1	PI3K/AKT	Proliferation, invasion, metastasis, cell survival
TGU1	miR-204–5p	JAK2	JAK2/STAT3	Growth, angiogenesis, invasion, metastasis, cell survival
/	miR-181b	SOCS2	JAK2/STAT5	Growth, angiogenesis, invasion, metastasis, cell survival
CRNDE	miR-203	VEGFA	VEGFA	Angiogenesis
SNHG1	miR-6838–5p	PIM3	PIM3/RhoA	Cell proliferation, migration, invasion
/	miR-21	ASPP2	P38	Apoptosis
NBR2	miR-22	TCF7	Glucose metabolic pathway	Proliferation
CDR1(circRNA)	miR-7–5p	KLF4	Stemness	Proliferation

Within this network, miRNAs act as strategic commanders exerting pleiotropic regulatory effects. The oncomiR miR-181b, by simultaneously suppressing phosphatase and tensin homolog (PTEN) and cell adhesion molecule 1 (CADM1), relieves the brake on the PI3K/AKT pathway and indirectly potentiates Wnt signaling, establishing a miR-181b–PTEN–AKT–GSK-3β–β-catenin positive feedback axis [69, 70]. On the other hand, it impairs cell adhesion and promotes invasion and metastasis, thereby cooperatively driving both “proliferation-survival” and “metastatic potential” malignant programs. Conversely, the tumor-suppressive miRNA miR-186 exerts restraining effects by targeting YAP1 and the autophagy-related gene ATG7, simultaneously inhibiting proliferative signaling and depriving cells of metabolic adaptability [71]. Furthermore, the lncRNA MIR205HG plays a significant role in this regulatory network: it competitively binds to miR-514a-5p, targeting MAPK9 and activating the MAPK signaling pathway [33]. Moreover, MIR205HG acts as a sponge for miR-205–5p, alleviating its suppression of the PI3K/AKT pathway and establishing an additional cooperative axis that promotes HB progression. These findings identify MIR205HG as a potential effective biomarker and novel therapeutic target.

In summary, the molecular architecture of HB constitutes a dynamic network with core pathways, such as the Wnt/β-catenin and PI3K/AKT pathways, as its “backbone”, and pleiotropic regulators, such as miR-181b and miR-186, and lncRNAs, such as MIR205HG, as its “control system”. This systems biology perspective not only provides novel insights into tumor heterogeneity and therapeutic resistance but also opens new avenues for clinical translation: specific miRNA and lncRNA expression signatures could serve as biomarkers for diagnosis and prognosis assessment, whereas RNA-based intervention strategies, such as restoring miR-186 function, inhibiting miR-181b activity, or targeting MIR205HG, hold promise as “network therapies” that simultaneously modulate multiple network nodes, potentially advancing HB treatment from conventional chemotherapy into a new era of precision medicine.

### Application potential of miRNAs in HB treatment

The application potential of miRNAs in HBs has gradually attracted the attention of researchers. The pathogenesis of HB is complex and involves the regulation of various noncoding RNAs. In recent years, studies have shown that miRNAs play important roles in the occurrence, development, diagnosis, and treatment of HB. By analysing the expression profiles of specific miRNAs, new biomarkers can be identified for the early diagnosis and personalized treatment of HB. Research indicates that miR-203 is downregulated in HB, and its loss is associated with tumor angiogenesis, cell migration, and increased invasiveness. miR-203 directly targets VEGFA, inhibits the proliferation and migration of tumor cells, and plays a negative regulatory role in angiogenesis in HB [16]. This finding not only provides new clues for the screening of biomarkers for HB but also offers theoretical support for related therapeutic strategies. miRNA-targeted therapeutic strategies show broad prospects in the treatment of HB. In recent years, studies have shown that by using miRNA mimics or inhibitors to regulate abnormal expression, the malignant phenotype of tumor cells can be effectively reversed. Dong *et al*. demonstrated that transfection with the precursor of miR-34a-5p significantly reduced the growth of HB tumors *in vivo* while decreasing microvascular density and the number of proliferating tumor cells, highlighting the role of miRNAs in tumor angiogenesis [72]. Some studies have shown that miR-492 can regulate the metastatic process of HB through CD44, suggesting that miR-492 may serve as a potential therapeutic target [73]. Reports indicate that miR-378a inhibits the proliferation and invasiveness of HB cells through its negative regulatory effects on its targets vascular endothelial growth factor receptor, platelet-derived growth factor receptor β, and c-Raf, and enhances the sensitivity of HB cells to sorafenib combined with chemotherapy.

Additionally, the tumor suppressor miR-26a-5p inhibits the proliferation and colony formation of HepG2 and HuH6 cells, indicating its potential value as a novel molecular target for HB [74]. In summary, miRNAs show great application potential in the treatment of HB. Through further research on the functions of miRNAs and their targets, combined with the accumulation of clinical data, new diagnostic tools and therapeutic methods to improve the prognosis of HB patients can be developed.

#### miRNAs as diagnostic and prognostic biomarkers

In the field of HB, the potential of miRNAs as prognostic biomarkers has gradually gained attention. Multiple studies have shown that specific miRNAs (such as miR-200c-3p, miR-222–5p, and miR-512–3p) are significantly associated with tumor stage and patient survival rates. For example, the expression level of miR-200c-3p in HB tissues and cell lines is closely related to patient prognosis, with higher levels of miR-200c-3p associated with better survival rates [75]. In contrast, high expression of miR-222–5p and miR-512–3p is associated with poor prognosis, suggesting that these miRNAs may become new prognostic markers to assist clinicians in assessing and predicting patient conditions. Surprisingly, we found that the expression of miRNAs in embryonic and fetal HBs was inversely correlated. For example, miR-18a is expressed at higher levels in adult livers than in embryonic livers, whereas miR-122 is expressed at higher levels in embryonic and fetal livers than in adult livers [76]. A cohort study of 22 HB patients revealed that the expression level of miR-17 in HB tumor samples was significantly reduced and associated with poorer prognosis. In this study, miR-19b was expressed at higher levels in the embryonic subtype than in the fetal subtype [77]. Another comparison of 32 HB samples with a control group revealed that the levels of exosomal and plasma miR-21 were significantly greater than those in the control group. Vascular invasion, the pretreatment extent of disease (PRETEXT) staging system, tumor metastasis, and exosomal miR-21 are independent risk factors affecting the event-free survival of HB patients [78, 79].

Similarly, our team discovered that the miR-181b/SOCS2/JAK2/STAT5 signaling axis shows that high expression of miR-181b in HB is positively correlated with tumor malignancy, promoting the activation of the JAK2/STAT5 signaling pathway by reducing SOCS2 expression levels, thereby driving the progression of HB. These findings suggest that some miRNAs may serve as potential prognostic biomarkers, aiding in the early diagnosis and treatment of HB [24]. For example, miR-492 is a potential biomarker in metastatic HB, and the overexpression of pleomorphic adenoma gene 1 (PLAG1) in HB, which significantly affects miR-492, as demonstrated through RNA interference analysis combined with miRNA array studies, has been characterized. Furthermore, miR-492 can originate from the coding sequence of the HB marker gene keratin 19 (KRT19). In metastatic HB tumor samples, significantly elevated levels of coexpressed KRT19 and miR-492 were detected [78]. Interestingly, since metastatic HB is often associated with poor prognosis, Wu *et al*. reported in a meta-analysis that the dysregulation of miRNAs (miR-34a, miR-34b, miR-34c, miR-21, miR-222, miR-224, and miR-193a) is related to poorer overall survival in HB patients, and the abnormal expression of miRNAs (miR-21, miR-492) is associated with poor prognosis in children with HB. These findings suggest that miRNAs may be potential biomarkers for distinguishing HB patients from healthy individuals, providing important evidence for the development of future noninvasive diagnostic methods for HB [21].

Additionally, studies have shown that high expression of insulin-like growth factor 2 (IGF2)-derived intronic miR-483 can predict the prognosis of HB patients and is closely related to vascular invasive growth in HB patients [80]. Overall, miRNA-targeted therapeutic strategies can effectively reverse the malignant phenotype of HB by regulating the interactions between key lncRNAs and their target genes. Future research should continue to explore the clinical application potential of these targeted therapies and further deepen the understanding of their molecular mechanisms to advance the process of personalized treatment for HB.

#### Synergistic role of miRNA and alpha-fetoprotein: moving beyond classical biomarkers

Alpha-fetoprotein (AFP) has long been an indispensable serological biomarker for the diagnosis and prognosis of HB. However, while AFP is sensitive, its specificity is limited. Elevated AFP levels can also occur during physiological peaks in infancy, benign liver diseases (viral hepatitis, cirrhosis), and certain germ cell tumors, potentially leading to false-positive results [81, 82]. More critically, a small but significant subset of HB patients, often with specific histological subtypes, present with low or normal AFP levels, posing considerable challenges for diagnosis and monitoring [83]. In contrast, a study by Liu *et al*. involving 32 Chinese subjects revealed that the concentration of exosomal miR-21 in the HB group was significantly greater than that in the exosome-depleted supernatant and whole plasma. Compared with that in controls, the expression of miR-21 in both plasma and exosomes was markedly elevated in HB patients [11]. For HB diagnosis, exosomal miR-21 demonstrated significantly superior accuracy to that of the AFP level. Growing evidence indicates that specific miRNA profiles are strongly correlated with AFP, and their combination can provide more robust clinical information, enabling more precise early diagnosis and subtype differentiation [84, 85].

Dynamic monitoring of AFP is a cornerstone for assessing treatment response and predicting recurrence in HB patients. However, the decline in AFP levels can be delayed and does not distinguish between molecular subtypes driven by different genetic alterations. Research data show that miR-181b directly promotes tumor progression by activating the PI3K/AKT and Wnt/β-catenin pathways [69]. Consequently, high pretreatment levels of miR-181b may indicate resistance to conventional chemotherapy or a more aggressive disease phenotype, which is associated with poor prognosis. During therapy, persistently high expression or an insignificant early decline in miR-181b might serve as an earlier warning of a suboptimal treatment response or minimal residual disease than AFP monitoring alone. Conversely, restored expression of miR-186 may correlate with favorable treatment response and prognosis. Therefore, integrating miRNA expression profiles with AFP levels can facilitate the construction of a multidimensional prognostic assessment system.

In conclusion, combining miRNA biomarkers with classical AFP testing represents a future direction for precision medicine in HB. This integrated strategy not only compensates for the limitations of AFP in specific clinical scenarios, thereby enhancing diagnostic accuracy, but also enables more refined risk stratification, earlier assessment of treatment response, and more timely prediction of recurrence by providing independent molecular pathological information. Future large-scale prospective studies designed to validate the clinical utility of such a combined miRNA–AFP signature model will be crucial for translating this approach from the laboratory to clinical practice.

#### Relationships between miRNAs and drug resistance

**Figure 4.**
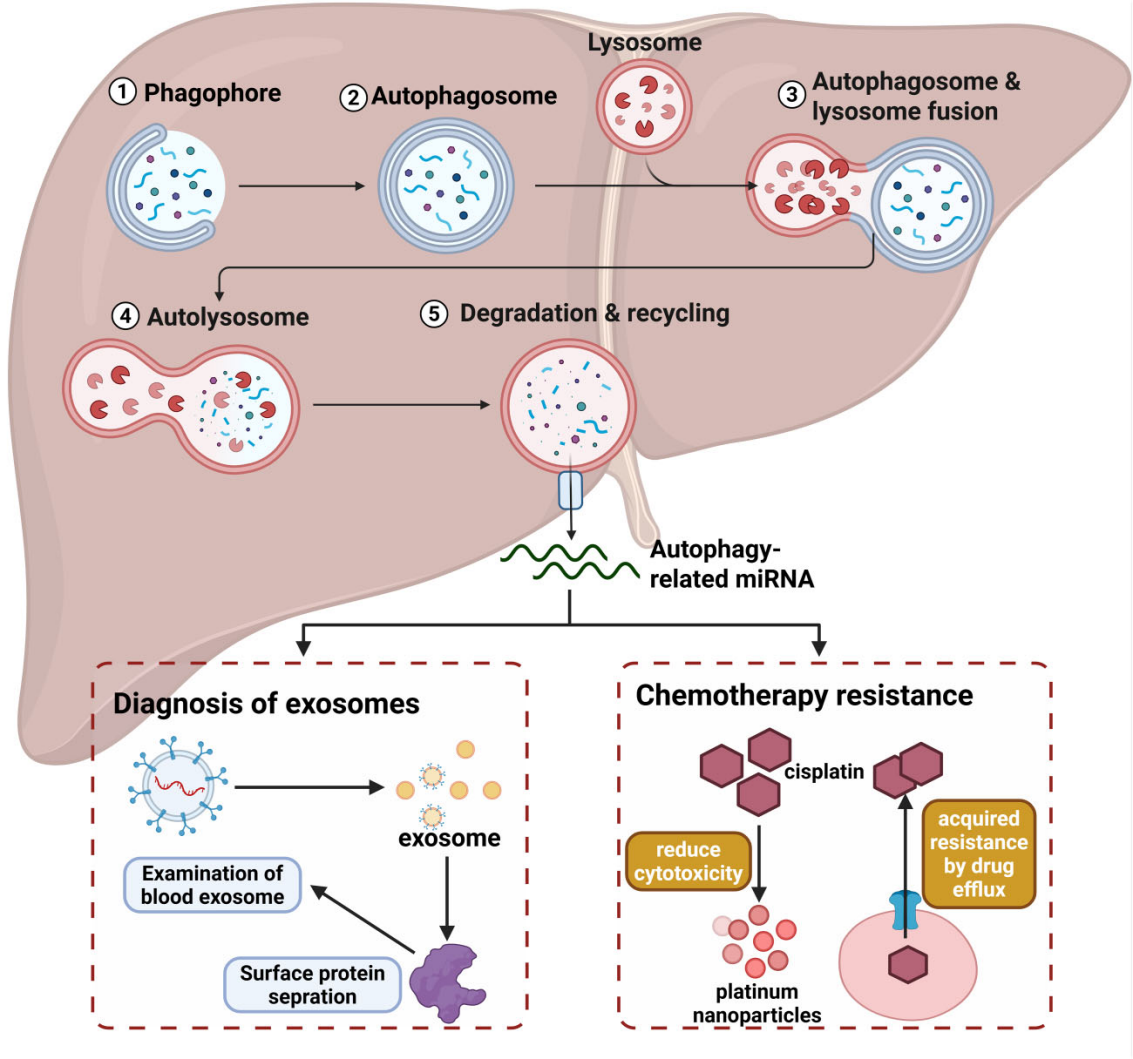
The role of autophagy-related miRNAs in HB. miRNAs enhance therapeutic effects by regulating autophagy, increasing tumor sensitivity to cisplatin, and reducing resistance, thereby inhibiting tumor growth. The mechanisms may include the following. (i) Inhibition of prosurvival autophagy: tumor cells often use autophagy as a “shield” to evade the cytotoxic effects of chemotherapy drugs. Downregulating certain miRNAs that promote protective autophagy (or upregulating miRNAs that inhibit such autophagy) can weaken this defense mechanism in tumor cells, increasing the effectiveness of drugs in inducing apoptosis. (ii) Impact on drug transport and metabolism: miRNAs may influence the accumulation and efficacy of cisplatin in cells by regulating genes involved in drug uptake, efflux, or metabolism (such as transport proteins). (iii) Regulation of DNA damage repair: cisplatin exerts its cytotoxic effects by causing DNA damage. miRNAs can enhance the cytotoxic effect of cisplatin by affecting DNA damage repair pathways, leading to the accumulation of damage. (iv) Synergistic induction of cell death: by regulating the cross-talk between autophagy and other cell death pathways (such as apoptosis and necroptosis), miRNAs may promote a synergistic effect on cell death. These actions collectively lead to a reduction in tumor cell resistance to cisplatin, significantly improving the efficacy of chemotherapy. Additionally, exosomal miRNAs related to autophagy are expected to become new targets for the diagnosis and treatment of HB.

Chemotherapy resistance is a complex phenomenon in which miRNAs play a role in the progression of various malignancies, including in chemotherapy resistance, by interacting with and inhibiting the expression of targeted mRNAs. Autophagy is a highly conserved process that involves the degradation of cytoplasmic contents and organelles via lysosomes to cope with environmental stresses such as hypoxia and starvation. Autophagy contributes to the response to chemotherapy: it can act as a protective mechanism mediating resistance to chemotherapy or can induce autophagic cell death and mediate sensitivity to chemotherapy [86]. The relationship between miRNAs and drug resistance in HBs has received increasing attention (Fig. 4). Studies have shown that the resistance of HB cells to chemotherapeutic drugs such as cisplatin and targeted drugs such as sorafenib is closely related to the expression of various miRNAs. miRNAs influence the biological characteristics of HB cells by regulating key signaling pathways, thereby altering their sensitivity to drugs. Research on miR-25 in HCC indicates that high expression of this miRNA is associated with chemotherapy resistance. As the regulatory role of miRNAs in autophagy and tumor development is further understood, the potential of miRNAs in cancer treatment and diagnosis is gaining increased attention [87]. miR-25 promotes autophagy by inhibiting the expression of F-box and WD repeat domain containing 7 (FBXW7), thereby increasing the resistance of HCC cells to sorafenib [88]. Additionally, the lncRNA H19 has been shown to promote the resistance of HB cells to sorafenib by increasing the expression of miR-675 [89]. In terms of resistance to cisplatin, circRNAs and lncRNAs regulate cell resistance through interactions with miRNAs. For example, circ_RBM23 is upregulated in cisplatin-resistant HCC and promotes cell resistance to cisplatin by regulating the interaction between miR-338–3p and RAB1B [90]. Similarly, research on lncRNA KCNQ1OT1 in colorectal cancer has shown that this lncRNA promotes cancer cell resistance to cisplatin by interfering with miR-125b-5p [91]. Fu *et al*. reported that the expression of miR-378a is significantly reduced in tissue samples from liver cancer patients, which inhibits the proliferation and invasion of HCC and HB cell lines [74]. It can suppress the growth of liver cancer cells and increase their sensitivity to sorafenib-based chemotherapy. Serine/threonine kinases and matrix metalloproteinase 2 are regulated solely by miR-378a, with a more pronounced regulatory effect when miR-378a is combined with sorafenib.

Moreover, studies have shown that miR-30e-3p, miR-30d, miR-31–5p, and miR-221 are associated with sorafenib resistance in renal and liver cancer cells [92–94]. Additionally, sorafenib increased the expression of miR-27a-3p, miR-122–5p, miR-193b-3p, miR-375, and miR-505–5p in HepG2 cells but decreased the expression of miR-148b-3p, miR-194–5p, miR-200c-3p, miR-222–5p, miR-512–3p, and miR-551a [75]. A recent study revealed differential lncRNA‒miRNA regulatory axes in HepG2 and SNU449 liver cancer cells. These effects led to increased expression of mRNAs in HepG2 and SNU449 cells under sorafenib treatment, which have different therapeutic effects [95]. These altered regulatory networks resulted in distinct patterns of mRNA expression in the two cell lines. These studies indicate that miRNAs play important roles in drug resistance in HBs, potentially through the regulation of cell autophagy, signaling pathways, and cellular metabolism.Future research could explore the potential of these miRNAs as therapeutic targets to overcome chemotherapy resistance in HB and improve treatment outcomes. In summary, these miRNAs and their associated lncRNAs and circular RNAs provide an important molecular basis for developing novel therapeutic strategies that can specifically enhance sensitivity to chemotherapy drugs, thereby improving the clinical prognosis.

## Conclusions and future directions

Despite some progress in the study of HBs in recent years, many challenges remain. Future research directions will focus on the molecular mechanisms of HB, early diagnosis, and targeted therapies. First, early diagnosis of HB is an important research direction. Existing diagnostic methods still have certain limitations, especially in the early stages of tumors. Therefore, developing new biomarkers and imaging technologies will be a key task in future research. By identifying the specific miRNAs dysregulated in HB, the accuracy of early diagnosis can be improved, thereby enhancing patient prognosis. Second, with the development of targeted therapies and immunotherapies, treatment strategies for HB should also be continuously optimized. Future research should explore the potential of targeted therapies involving miRNAs and their corresponding signaling pathways in combination with clinical trials to assess their efficacy and safety. Moreover, the use of new drug combination strategies also needs to be emphasized to improve the effectiveness of existing therapies. Finally, interdisciplinary collaboration will play an important role in future HB research. The integration of basic research and clinical studies will promote a comprehensive understanding of HB and drive the development of new therapies. By integrating bioinformatics, translational medicine, and clinical trials, researchers can better identify biomarkers, mechanisms, and treatment strategies for HB, thereby improving patient survival rates and quality of life. In this process, data sharing and multicenter studies will also be key factors in enhancing research efficiency and the reliability of results. In summary, future research on HBs will focus on deeper exploration of molecular mechanisms, development of early diagnostic biomarkers, optimization of targeted treatment strategies, and strengthening interdisciplinary collaboration. These studies will help advance the clinical management of HB, providing patients with better treatment options and prognoses.

In recent years, research on miRNAs in HB has increased, especially in clinical translational studies, demonstrating their great potential as liquid biopsy markers. The development of highly sensitive miRNA detection technologies is key in this field. Studies have shown that miRNAs such as miR-200c and miR-21 play important roles in the pathogenesis of HB and that their expression levels in liquid biopsies are closely related to clinical prognosis. Specifically, miR-200c is highly expressed in HB cells, and its detection in peripheral blood and extracellular vesicles provides new ideas for the early diagnosis of HB [96, 97]. By employing highly sensitive detection technologies, changes in these miRNAs in the tumor microenvironment can be captured more accurately, providing more reliable diagnostic evidence for clinical use. In addition, breakthroughs in the design of miRNA-targeted drugs and nanodelivery systems have been made in the treatment of HB. Researchers have developed various nanoparticle systems for the effective delivery of miRNAs to HB cells. For example, via the use of poly (lactic-co-glycolic acid)-PEG (PLGA-PEG) nanoparticle carriers, miR-130a-5p can be targeted and delivered to HB cells, significantly inhibiting tumor cell proliferation and increasing sensitivity to chemotherapy drugs [98]. This nanodelivery system not only improves the bioavailability of miRNAs but also reduces the systemic toxicity of chemotherapy drugs, highlighting its future application prospects in HB treatment. By combining miRNA nanodelivery systems with targeted drugs, researchers have made significant progress in improving treatment efficacy and safety. In summary, miRNAs show great potential in clinical translational research for the treatment of HB. In the future, with continuous advancements in detection technologies and nanodelivery systems, miRNAs may become important components of early diagnosis and targeted therapy for HB, providing patients with more precise treatment options.

In the application of miRNA therapy, technical and safety issues are key challenges. To optimize miRNA delivery carriers, researchers have adopted various strategies to reduce off-target effects and immune responses, ensuring the safety of clinical applications. Specifically, by utilizing novel delivery systems such as nanoparticle carriers, liposomes, and polymer gene carriers, researchers can increase the bioavailability of miRNAs while minimizing their impact on normal cells [99]. For example, liposomes and polymer carriers can effectively protect miRNAs from degradation by enzymes *in vivo* while enhancing their cellular uptake and targeting effects, thereby improving their therapeutic efficacy. Additionally, to address off-target effects in miRNA therapy, researchers are also committed to developing more precise delivery systems. By designing and optimizing miRNA sequences, combined with specific guiding RNA technologies, the selective binding of miRNAs to target genes can be improved, reducing interference with nontarget genes. For example, inhibitors of miR-21 have been shown to effectively reduce the proliferation of tumor cells while having relatively little impact on normal cells, indicating that by designing specific delivery systems, side effects can be minimized while treating cancer [100]. With respect to immune responses, studies have shown that miRNA delivery carriers may induce immune system reactions; therefore, researchers are exploring ways to reduce this response by modifying delivery carriers or combining them with immunomodulators. For example, the use of modified nanoparticle carriers can reduce immunogenicity, making treatments safer for clinical applications [101]. Moreover, combining gene editing technologies such as CRISPR-Cas9 can achieve precise regulation of miRNA-targeted genes at the molecular level, further reducing the impact on nontarget genes and increasing the safety and efficacy of treatments. Overall, addressing the technical and safety issues of miRNA therapy relies on interdisciplinary collaboration, including the close integration of materials science, molecular biology, and drug development. Future research should continue to focus on improving and optimizing delivery systems to ensure the effectiveness and safety of miRNA therapy in clinical settings while minimizing side effects and enhancing patients' quality of life. The implementation of these strategies will pave the way for the application of miRNAs in the treatment of cancers such as HB, promoting the development of personalized medicine.

Despite the exciting potential of miRNA-based “network therapy” for the precise treatment of HB and other pediatric diseases, its clinical translation still faces multiple challenges. There is a critical lack of pediatric-specific pharmacokinetic and safety data. The vast majority of preclinical studies rely on adult animal models, whereas children undergo dynamic development of their organs, metabolic enzymes, and immune systems. Data on the distribution, metabolism, excretion, and potential long-term toxicity of antisense oligonucleotides or miRNA mimics in pediatric populations are virtually absent. For example, a nanocarrier deemed safe in adult models may cause unforeseen off-target effects on the developing liver or nervous system. Second, delivery systems present significant bottlenecks: nanocarriers (lipid or polymer nanoparticles) can induce acute inflammatory responses, complement activation, and dose-limiting hepatotoxicity, which is a particular concern for patients with potentially compromised liver function. These toxicities may be amplified in children, and their long-term impacts remain entirely unknown. Additionally, synthetic RNA molecules and their delivery vehicles can be recognized by pattern recognition receptors (Toll-like receptors), triggering potent innate immune responses that may not only diminish therapeutic efficacy but also provoke severe systemic inflammation, posing life-threatening risks to pediatric patients. Furthermore, while the inherent multitarget nature of miRNAs forms the basis for their use as “network therapeutics,” it also raises the risk of off-target toxicity. For example, an antagonist designed to inhibit miR-181b might inadvertently affect unknown targets essential for normal hematopoiesis or neurodevelopment. Thus, comprehensive off-target assessments using whole transcriptomics in reliable pediatric disease models are imperative before advancing any clinical trials. In summary, future research must prioritize the establishment of robust pediatric preclinical models to address gaps in pharmacology and safety profiling while also advancing the development of safer, tumor-targeted next-generation delivery technologies. By openly addressing these challenges and defining them as current research priorities, the field can collectively work toward the safe and effective application of these promising molecular tools in pediatric clinical practice.

In conclusion, this review consolidates compelling evidence that miRNAs are pivotal regulators of the pathogenesis, progression, and therapeutic response of HB. They exert their influence through complex ceRNA networks by interacting with lncRNAs and circRNAs, thereby modulating key oncogenic signaling pathways such as the Wnt/β-catenin, MAPK, PI3K/AKT, and JAK2/STAT3 pathways. These interactions critically govern diverse aspects of HB malignancy, including tumor cell proliferation, metastasis, stemness, angiogenesis, and chemoresistance. The significant potential of specific miRNAs as noninvasive diagnostic and prognostic biomarkers is increasingly evident. Furthermore, emerging strategies utilizing miRNA mimics, inhibitors, and advanced nanoparticle-based delivery systems highlight promising frontiers for targeted HB therapy. Future research should focus on translating these mechanistic insights into clinical applications, leveraging interdisciplinary approaches to develop effective miRNA-based diagnostic tools and therapeutic interventions that can ultimately improve outcomes for children with HB.
